# Stress leads to prosocial action in immediate need situations

**DOI:** 10.3389/fnbeh.2014.00005

**Published:** 2014-01-22

**Authors:** Tony W. Buchanan, Stephanie D. Preston

**Affiliations:** ^1^Department of Psychology, Saint Louis UniversitySt. Louis, MO, USA; ^2^Department of Psychology, University of MichiganAnn Arbor, MI, USA

**Keywords:** stress, decision making, altruism, empathy, TSST, perception-action

## Abstract

Stress clearly influences decision making, but the effects are complex. This review focuses on the potential for stress to promote prosocial decisions, serving others at a temporary cost to the self. Recent work has shown altruistic responses under stress, particularly when the target’s need is salient. We discuss potential mechanisms for these effects, including emotional contagion and offspring care mechanisms. These neurobiological mechanisms may promote prosocial—even heroic—action, particularly when an observer knows the appropriate response and can respond to a target in need. The effects of stress on behavior are not only negative, they can be adaptive and altruistic under conditions that promote survival and well-being at the individual and group level.

## Introduction

When most people say that they are “stressed”, they are referring to a negative, subjective feeling of being overwhelmed by the amount of work or responsibilities facing them—an amount that may be too much to handle. In contrast, the biological term “stress” refers to an evolved, metabolic, neurohormonal response to unpredictability and uncontrollability, which prepares the organism for acute threat, centering around the coordinated action of the sympathetic nervous system and hypothalamic pituitary adrenocortical (HPA) axis (Mason, 1968a,b; Koolhaas et al., 2011). This stress response promotes adaptive action to real challenge and is not necessarily damaging unless chronic. Using this biological framework for understanding stress, we review recent and accumulating evidence that stress can actually promote prosocial behavior. Here, we define prosocial behavior as occurring whenever someone voluntarily and intentionally serves another at a temporary cost to the self (Eisenberg and Miller, 1987). While there are myriad effects of stress on behavior, we only briefly discuss those before focusing on the role of stress in prosocial behavior *per se*, which is not often appreciated and can be best understood through this functional, neurobiological approach.

## Stress and decision making

The physiological stress response evolved as an adaptive way to motivate behavior and release metabolic energy in situations of acute need (see Johnson et al., 1992; Sapolsky et al., 2000). For example, when an organism detects the presence of a predator, a stress response may be activated that temporarily delays longer-term processes like wound healing and digestion in order to marshal an active response like fighting or running away. Such stress-induced responses are not always aggressive, however, as heroic rescues provide dramatic examples of prosocial responses to stress and challenge.

The adaptive stress response includes both metabolic and behavioral changes (Pfaff, 2005). Stress-induced changes in behavior and metabolism often work together toward the same goal. For example, stress-induced vasopressin enhances thirst behavior while increasing water retention via the kidneys (Vander et al., 1998). Similarly, epinephrine release enhances attentional processes and accelerates the heart, both of which promote responsiveness in times of need (Mason, 1968b). While stress enhances the processing of needed resources like metabolic energy, it does so by temporarily reducing longer-term functions that are not immediately necessary, like digestion, growth, and immune function (see Johnson et al., 1992). This shift in metabolic and behavioral processing from building for the future, to focusing on the present emergency is adaptive for current survival but unsustainable in the long term. While most researchers emphasize the role of this shift towards immediacy for promoting aggressive or fearful behaviors, it may play a similar role in prosocial acts, particularly during emergencies, when people must act quickly to assist endangered others.

Much of the research examining the influence of stress on decision making in humans has focused on risk taking at the individual level, such as examining how someone’s stress may affect their decisions to risk money under changing probabilities of winning or losing. The consensus from this work is that stress does indeed affect decision making, but in a complex manner that is dependent upon the timing and type of decision as well as the age and gender of the decision maker (for reviews see Mather and Lighthall, 2012; Starcke and Brand, 2012). As an illustrative example of this complexity, in our own study examining the effects of stress on gambling, females made more advantageous choices after stress while males made more disadvantageous choices (Preston et al., 2007b). In another study, stressed participants performed more advantageously in a Game of Dice task when they were tested soon after the stressor, when the adrenergic system was most activated, but were less advantageous later when glucocorticoid levels were at their peak (Pabst et al., 2013).

Because results in this domain sometimes report more advantageous decisions under stress and other times report more disadvantageous decisions, it is too soon to make sweeping generalizations about the effects of stress on decision making. But generally, studies that examine stress effects on learning and memory find an increased reliance on automatic over controlled cognitive processes during stress (Schwabe et al., 2012). For example, stressed rodents and humans increasingly rely upon habitual behaviors, rather than more flexible cognitive strategies in a learning task (Schwabe et al., 2008; Schwabe and Wolf, 2009). Similarly in human decision making, stressed humans make quick, heuristic judgments rather than more contemplative ones (i.e., relying on System 1 over System 2, cf., Kahneman, 2011; see review in Starcke and Brand, 2012). Such habitual responses do not map neatly onto risk aversion or risk seeking (or advantageous vs. disadvantageous decisions), as one can habitually or intuitively make a conservative choice—such as sticking with a low-payoff, low-risk investment—or a very risky one—such as choosing to snatch a child from the path of an oncoming car. Across cases, however, stress appears to adaptively shift the focus of the individual on the immediate issue so that they can respond quickly while discounting longer-term problems that may be irrelevant at present, similar to the effects of stress on metabolic function.

There is also a considerable body of work demonstrating the effects of the social environment on decision making across species, particularly with regard to decisions in the context of scarcity (for review see van den Bos et al., 2013). While group living provides fitness benefits to both the individual, her relatives, and the group as a whole (Hamilton, 1964; West et al., 2007), group life also utilizes more resources than independent living and adds significant social stress from trying to balance needs across group members. Interactions with conspecifics are among the most unpredictable and uncontrollable situations that an organism can encounter (for review see Buchanan et al., 2009)—characteristics that are central to the initiation of a physiological stress response (Mason, 1968a,b; Koolhaas et al., 2011). For example, disputes over shared resources can lead to potentially lethal aggression against rivals (Potegal and Knutson, 1994; Anderson and Bushman, 2002; Miczek et al., 2007). However, just as the effects of stress on decision making in general do not only predict simple, unidirectional outcomes, stress in a social group context can also foster affiliative responses, depending on what is more adaptive for the individual in context. For example, studies of crowding in nonhuman primates found that, contrary to the assumption that crowding unilaterally produces violence and aggression, monkeys and apes actually increase affiliative gestures and grooming when crowded to compensate for the challenges of reduced space (de Waal et al., 2000).

## Stress can have prosocial consequences

A growing body of research describes situations where stress actually promotes prosocial behaviors like empathy and altruism (Taylor et al., 2000; de Waal and Suchak, 2010). Here we review some of key findings and discuss potential mechanisms to provide a counterpoint to the prevailing view of stress as only promoting aggressive or negative effects on health, brain function, and decision making (Shirtcliff et al., 2009).

Males often exhibit a “fight-or-flight” response to stress, but females across many species may be better characterized by a more prosocial “tend and befriend” response (Taylor et al., 2000; Preston, 2013). Tending, meaning caring for offspring, and befriending, meaning connecting with conspecifics, protects an organism’s offspring and may help social groups collaborate under stressful conditions (Taylor et al., 2000). Several recent studies have supported the concept that stress can lead to prosocial behavior, even in males (Takahashi et al., 2007; von Dawans et al., 2012; Vinkers et al., 2013). In one study, healthy young men were exposed to a stressful task (the Trier Social Stress Test) or a nonstressful comparison task, followed by measures of pro-social and anti-social behavior. The stress condition increased subsequent trust, trustworthiness, and sharing, without changing punishment or non-social risk taking (von Dawans et al., 2012), undermining the assumption that stressed men would revert to anti-social “fight-or-flight” responses and supporting the possibility of prosocial effects. In another study, people were more prosocial when they decided more quickly (Rand et al., 2012), indicating that even a shift towards more automatic responses—as occur under stress—can produce prosocial outcomes. In another study, Vinkers et al. (2013) showed greater altruistic punishment in the Ultimatum Game when participants were tested immediately after a stressor (rather than 90 min after stress onset), demonstrating a greater tendency to punish non-cooperators in the early phase of the stress response. Stress induced by the Trier Social Stress Test (TSST) did not lead to increased selfish responses in a moral decision making task in young healthy men and women, but those individuals who showed a greater stress-induced cortisol response made more selfish decisions (Starcke et al., 2011). By contrast, participants who reported greater positive affect in response to the TSST made more altruistic decisions. These results demonstrate how individual differences in physiological and psychological responses to stress may push decision making from self-less to selfish. Individuals who find a stressor to be more of a challenge (and report more positive affect, Lazarus and Folkman, 1984) may respond to another in need in a more prosocial manner. This is in contrast to individuals who find a stressor to be a threat (and produce more of a cortisol response). These individuals may respond to others in a more selfish manner.

## Possible mechanisms for stress-induced altruistic aid

Preston et al. (2007a,b) have extended the known neural mechanisms required for caring for and retrieving helpless offspring in rats, mice, and monkeys to the context of human aid (Brown et al., 2012; Preston, 2013). The neural circuits supporting offspring care and retrieval largely overlap with those generally associated with motivational decision and reward processes in the mammalian mesolimbocortical system, including the amygdala, orbitofrontal cortex, nucleus accumbens, and downstream motor regions as well as neurohormones like dopamine, oxytocin, and the hormones of pregnancy and parturition (see also Preston and de Waal, 2011). These circuits act in concert to shift responses to vulnerable neonates from avoidance to approach as a result of the birth process or passive habituation (for virgin females and even for males), with a critical role for the medial preoptic area of the hypothalamus in retrieval *per se* (Lonstein and Morrell, 2007; Numan, 2011). Thus, these systems promote action towards one’s own offspring, but can also promote active retrieval and protection of unrelated, distressed offspring, even in nonparents.

By extending this neural system for offspring care to human altruism—helping unrelated individuals at a current cost to the self—one can perhaps understand seemingly reckless acts of heroic human altruism under stressful conditions, as when people rush into burning buildings, icy waters, or onto subway tracks to save complete strangers. These emergency situations, and many less dramatic or potentially lethal cases, inherently involve a high level of stress in the target in need as well as surrounding observers. Despite this stress—and perhaps even because of it (we would argue)—people do sometimes make an immediate and risky decision to help. Of course, people also often respond selfishly or apathetically to those in need in these stressful situations, as shown in bystander apathy research (Darley and Latane, 1968; Latane and Darley, 1969). But theory and research suggest that these dichotomous responses can be mapped on to the avoidance vs. approach systems of rodent offspring care in a way that predicts that humans will avoid helping when they are fearful and/or lack the necessary response but will “rush in” when they feel confident and know what to do (see Preston, 2013 for details). Thus, some of the most compelling human examples of altruism—heroic ones where someone risks their very life to save a complete stranger—can take place in the context of acute stress and are enacted for the benefit of another, even unrelated individual.

This offspring care framework for understanding altruism does not assume that stress evolved to either promote or prevent prosocial responding *per se*. Rather, the assumption (outlined in Preston and de Waal, 2011; Preston, 2013) is that the nervous system of altricial mammals evolved to attend to and respond to cues of offspring distress or vulnerability, at a benefit to inclusive fitness (cf., Hamilton, 1964). In addition, the stress response evolved independently but in tandem to secure metabolic resources for any immediate threat or need, at a benefit to one’s direct fitness. Once in place, these systems became motivationally independent of their origin and thus could be activated in group contexts under highly similar conditions to offspring care, even for unrelated or adult targets or abstract situations of need. It is assumed that even costly giving to strangers is adaptive or at least not selected against because the inherent approach-avoidance mechanism protects against responding when it endangers the giver or when the target does not have a real need. Moreover altruism promotes cooperation in group life and those highly dangerous acts of help are rare enough to limit the effects on reproductive success and also entail some benefits of their own to the giver (i.e., the rewards of heroism).

### Perception-action processes suggest affective contagion

Perception-action models of emotion (Preston and De Waal, 2002; Gallese et al., 2004; Singer and Lamm, 2009) suggest that the mere attended perception of another’s affective state spontaneously activates part of the observer’s own neural representations for feeling that state (see Preston and Hofelich, 2012 for a detailed explanation). This shared representational space is thought to be a byproduct of the way the nervous system evolved to process others’ actions and states, by making efficient use of representations that were already encoded and produced sensible action even when activated by others’ states. In salient cases, activating shared representations for emotions like distress produces visible or subjectively-felt contagion, as we have all experienced during a particularly dramatic movie when we feel a great loss when someone close to the lead character dies or when we wince in pain when he is injured. However, even when these phenomena are not subjectively experienced, at least part of the observer’s representations for feeling the observer’s state are assumed to be activated by our attended perception of him. This neural-level shared activation allows observers to cognitively understand how the other feels, which permits labeling their emotion and selecting the appropriate response (see also Preston and Stansfield, 2008; Buchanan et al., 2010; Hofelich and Preston, 2011). Thus, just as observing another person swinging a hammer activates part of your motor system that you use when swinging a hammer (Grafton et al., 1996), observing someone in pain (Singer et al., 2004) or imagining someone who is angry or afraid (Preston et al., 2007a) will activate common neural patterns developed through your personal past experiences with pain, anger, or fear.

If neural systems are generally designed in this perception-action manner, then it follows that observing another under stress could also activate observers’ own representations for experiencing stress, with consequent downstream hormonal changes in more salient cases. However, the case is less straightforward for stress as for pain or emotions like anger or fear. Biological stress is by definition an internal state, with unclear overt cues or behaviors that can be directly witnessed. Moreover, stressed participants themselves do not always know when they are stressed, as people often report that they were “distressed” or “scared” without producing a correlated, pronounced salivary cortisol response (Buchanan et al., 1999; Hellhammer and Schubert, 2012). However, if we can demonstrate that stress is contagious between individuals, then this could provide a proximate mechanism for promoting aid in contagiously stressed observers.

### The Empathic Trier Social Stress Test (eTSST)

Beyond physical emergencies, we often observe stress in others. What happens to us during these encounters is as varied as our numbers, but a few predictions can be made from the existing literature, suggesting that observers can contagiously catch the stress of targets, which can in turn promote prosocial aid (at least when there is a clear effective action that the observer can enact).

In our research we first wanted to confirm that physiological stress could resonate between humans (Buchanan et al., 2012). We used the gold standard of laboratory stress in humans: the Trier Social Stress Test (TSST; Kirschbaum et al., 1993; Dickerson and Kemeny, 2004). In the typical TSST, physiological measures such as salivary alpha amylase (sAA; an index of autonomic function) and cortisol are collected from the person who undergoes the public speaking and mental arithmetic tasks before observing experimenters. In our “empathic TSST” (or eTSST), we collected sAA and cortisol from the observing experimenters (hereafter “observers”) as well as the stressed speakers. To assess the role of trait empathy in this physiological stress resonance, observers also completed the Interpersonal Reactivity Index (IRI; Davis, 1983). The observers did indeed produce resonant cortisol reactivity in response to viewing stressed speakers, responses that were proportional to that of their paired speakers and not influenced by gender (e.g., male observers did not secrete more cortisol in response to female compared to male speakers). Further, observers’ trait empathy (assessed with the IRI) was positively correlated with their cortisol and sAA responses to viewing the stressed speaker.

The resonant cortisol response that we measured is importantly not just a shared form of arousal, as the cortisol response is not elicited by simple arousal (Lundberg and Frankenhaeuser, 1980; Lovallo et al., 1985), but requires combined feelings of uncontrollability and social evaluation (Dickerson and Kemeny, 2004; Koolhaas et al., 2011). The behavioral “stress” signals from the speakers that were detected by observers to produce the resonating stress response are topics for future research. The Perception Action Model of empathy (Preston and De Waal, 2002) predicts that observers centrally process a variety of multimodal cues (e.g., speech pitch, frequency, and content, facial expressions, posture) as a dynamic pattern that naturally activates neural patterns of activity in the observers, including their personal representations of stress from personal past experience. More empathic observers may show more physiological resonance with a speaker because they attend more closely to the speaker’s behavior across channels (Hofelich and Preston, 2011). Our current and future research examines the speaker cues associated with resonating stress in observers and any other observer characteristics that may contribute.

Future research should address whether this contagious or empathic stress response in observers of stress will also predispose observers to help. The offspring care model of altruism (Preston, 2013) predicts that observers who become contagiously activated by the immediate plight of a target should be more likely to help, just as contagious stress is thought to promote rescue behaviors in ants (Nowbahari et al., 2009). However, spontaneous prosocial responses are expected when an immediate response that the observer can enact is required. It is unclear what the “response” would be in the TSST, other than maybe offering reassuring feedback—a possibility that we are currently examining.

## Concluding remarks

A number of factors influence whether a stressed individual will act in a prosocial, ambivalent, or antisocial manner towards others. Research and theory suggest that individuals will actually respond prosocially during stress when the target is vulnerable, distressed, socially bonded or interdependent and when the observer does not fear for their own safety or security, does not have conflicting personal goals, and knows what needs to be done (Preston and De Waal, 2002; Preston, 2013). We have demonstrated that stress can be contagiously caught from targets to observers. Moreover, existing empirical data suggests that stress can promote prosocial behavior in some situations. These findings support the assumption that the target and their need must be salient to drive the response as, for example, stressed participants in modified dictator games donated more when they had interacted with the target (von Dawans et al., 2012) but not when it was a charitable organization (reviewed in van den Bos et al., 2013; Vinkers et al., 2013). Researchers will need to measure helping behavior as well as stress physiology in a much wider variety of contexts than simply donating money. The eTSST provides one avenue for studying this phenomenon in an ecological situation, but we also need paradigms that include a real ability for the observer to help the target in the moment of need—a feature that is not intrinsic to the TSST situation but is important in the theoretical offspring care framework. In addition, research in additional species on the extent to which aid is delivered under conditions of stress is needed—particularly to determine if only social species that live in extended groups show this behavior. With additional research, across species and domains, we can provide a more complete picture of how stress guides our decisions for good and for ill.

## Author contributions

Tony W. Buchanan wrote the first draft of the paper, supervised the preparation of the manuscript, and contributed intellectual and practical work to the paper. Stephanie D. Preston edited drafts and contributed intellectually and practically to the paper. Both authors contributed equally to this work.

## Conflict of interest statement

The authors declare that the research was conducted in the absence of any commercial or financial relationships that could be construed as a potential conflict of interest.
